# Spatiotemporal integration of tactile information in human somatosensory cortex

**DOI:** 10.1186/1471-2202-8-21

**Published:** 2007-03-14

**Authors:** Zhao Zhu, Elizabeth A Disbrow, Johanna M Zumer, David J McGonigle, Srikantan S Nagarajan

**Affiliations:** 1 Biomagnetic Imaging Laboratory, Department of Radiology, University of California, San Francisco, San Francisco, CA, 94143-0628, USA; 2 Center for Neuroscience, University of California, Davis, Davis, CA, 95616, USA; 3 Department of Neurology, University of California, Davis, Davis, CA, 95616, USA; 4 Center for Functional Imaging Studies, Western General Hospital, University of Edinburgh, Edinburgh, Scotland, EH4 2XU, UK

## Abstract

**Background:**

Our goal was to examine the spatiotemporal integration of tactile information in the hand representation of human primary somatosensory cortex (anterior parietal somatosensory areas 3b and 1), secondary somatosensory cortex (S2), and the parietal ventral area (PV), using high-resolution whole-head magnetoencephalography (MEG). To examine representational overlap and adaptation in bilateral somatosensory cortices, we used an oddball paradigm to characterize the representation of the index finger (D2; deviant stimulus) as a function of the location of the standard stimulus in both right- and left-handed subjects.

**Results:**

We found that responses to deviant stimuli presented in the context of standard stimuli with an interstimulus interval (ISI) of 0.33s were significantly and bilaterally attenuated compared to deviant stimulation alone in S2/PV, but not in anterior parietal cortex. This attenuation was dependent upon the distance between the deviant and standard stimuli: greater attenuation was found when the standard was immediately adjacent to the deviant (D3 and D2 respectively), with attenuation decreasing for non-adjacent fingers (D4 and opposite D2). We also found that cutaneous mechanical stimulation consistently elicited not only a strong early contralateral cortical response but also a weak ipsilateral response in anterior parietal cortex. This ipsilateral response appeared an average of 10.7 ± 6.1 ms later than the early contralateral response. In addition, no hemispheric differences either in response amplitude, response latencies or oddball responses were found, independent of handedness.

**Conclusion:**

Our findings are consistent with the large receptive fields and long neuronal recovery cycles that have been described in S2/PV, and suggest that this expression of spatiotemporal integration underlies the complex functions associated with this region. The early ipsilateral response suggests that anterior parietal fields also receive tactile input from the ipsilateral hand. The lack of a hemispheric difference in responses to digit stimulation supports a lack of any functional asymmetry in human somatosensory cortex.

## Background

The spatiotemporal integration of tactile inputs from different skin regions and across body parts is an important function of human somatosensory cortex. For example, the integration of inputs across the digits is vital for the successful manual manipulation and identification of objects. However, the mechanism of this integration is not well understood. Good candidates for the performance of this function are the second somatosensory area, S2, and the parietal ventral area, PV. These two fields are mirror symmetric representations of the body's surface [1-4], joined at the representation of the hand. Because it is difficult to distinguish between the hand representations of S2 and PV using functional imaging techniques we refer to this region as S2/PV.

Electrophysiological recording studies in monkeys indicate that neuronal receptive fields in S2 [2,5,6] and PV [2,4,7] are large, encompassing multiple digits or even the entire hand. Further, these receptive fields are often bilateral, including, for example, both the contra- and ipsilateral hand. Studies of neuroanatomical connections also show that, beside the local homotopic connections in the ipsilateral hemisphere, both S2 [1,7] and PV [1,4,7] have dense bilateral connections.

There is also evidence from human imaging studies that inputs from different skin regions interact in S2/PV. For example, bilateral integration of inputs from the hands takes place in human S2/PV [8-12]. In addition, the activation in human S2 evoked by stimulation of one finger can be modulated by simultaneous [11] or non-simultaneous [8,9,12] stimulation of other fingers of the same hand.

Another possibility is that integration of inputs from different skin regions takes place, at least to some extent, in anterior parietal areas 3a, 3b, 1 and 2 [9,11-15]. Previous MEG studies have confirmed that tactile stimulation to the human finger evokes responses in both contralateral anterior parietal fields as well as bilateral S2/PV [16,17]. Each finger has a distinguishable somatotopic representation in contralateral anterior parietal cortex [18,19]. However, there is some evidence that interaction among digit representations may also occur in anterior parietal cortex. For example, the strength of the early response to stimulation of a single finger was attenuated by simultaneous [11,14,15] or non-simultaneous stimulation of another finger at short (<100 ms) ISIs [13]. Further, the magnitude of spatial integration decreased with the distance of separation of the digit representations in anterior parietal fields [11,14]. There is also evidence from electrophysiological recording [20] and optical imaging [21] studies in monkeys that simultaneous stimuli from different skin regions could be merged together into a single activation zone in anterior parietal cortex.

However, in contrast to S2/PV, human MEG studies suggest that responses in anterior parietal cortex to stimulation of one hand are not affected by stimulation of the opposite hand [10-12]. This finding is supported by neuroanatomical results in monkeys indicating that the hand representation in 3b is largely acallosal [1,22-25]. Previous MEG work also suggests that the spatial integration of inputs across the digits is much stronger in S2/PV than in anterior parietal fields [9,11,12]. However, the extent of spatial integration in S2/PV, and the difference in extent and timing of integration between anterior parietal fields and S2/PV has not been quantified. These differences may represent different steps in the complex process of spatiotemporal integration.

In this study, we measured somatosensory evoked fields (SEFs) during non-simultaneous tactile stimulation of digits of both hands with an oddball paradigm. We compared the extent of spatial integration in the hand representations in human S2/PV and anterior parietal fields, and discuss the difference in the timing of integration in these two areas.

## Results

### Contra- and ipsilateral responses to index finger stimulation

#### 1). Dipole orientation and localization

**Figure 1:**
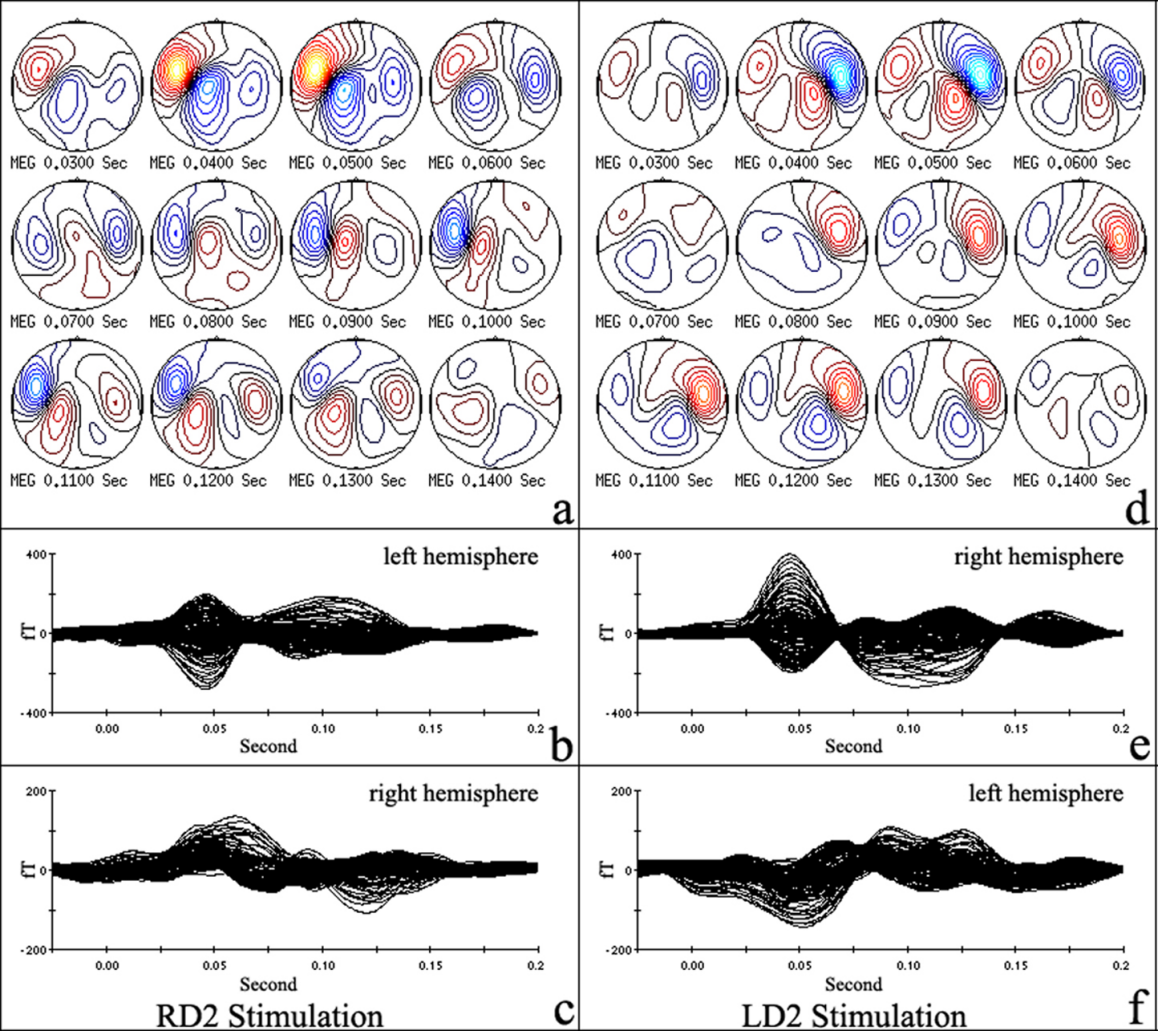
**Time courses of MEG maps and waveforms**. Averaged responses to index finger tactile stimulation at low rate (stimulation condition 1 in Figure 6; the strongest response was observed under this condition) were recorded from a right-handed subject. The left and right columns show responses to RD2 and LD2 stimulation, respectively. The two top (**a **and **d**) panels represent the series of contour plots of sensor data showing the time course of the averaged evoked magnetic fields. Each plot shows the sensor data interpolated between the 275 sensors at different latencies. The nasion is pointing up, the right ear is to the right, and the left ear is to the left (top view). Panels b, c, e and f show averaged evoked magnetic field responses; each line depicts an average of the data from a single sensor over all trials for one condition. Panels **b **and **e **show response waveforms recorded from half of all sensors over the hemisphere contralateral to the stimulated index finger, **c **and **f **from the hemisphere ipsilateral to the stimulated index finger.

**Figure 2:**
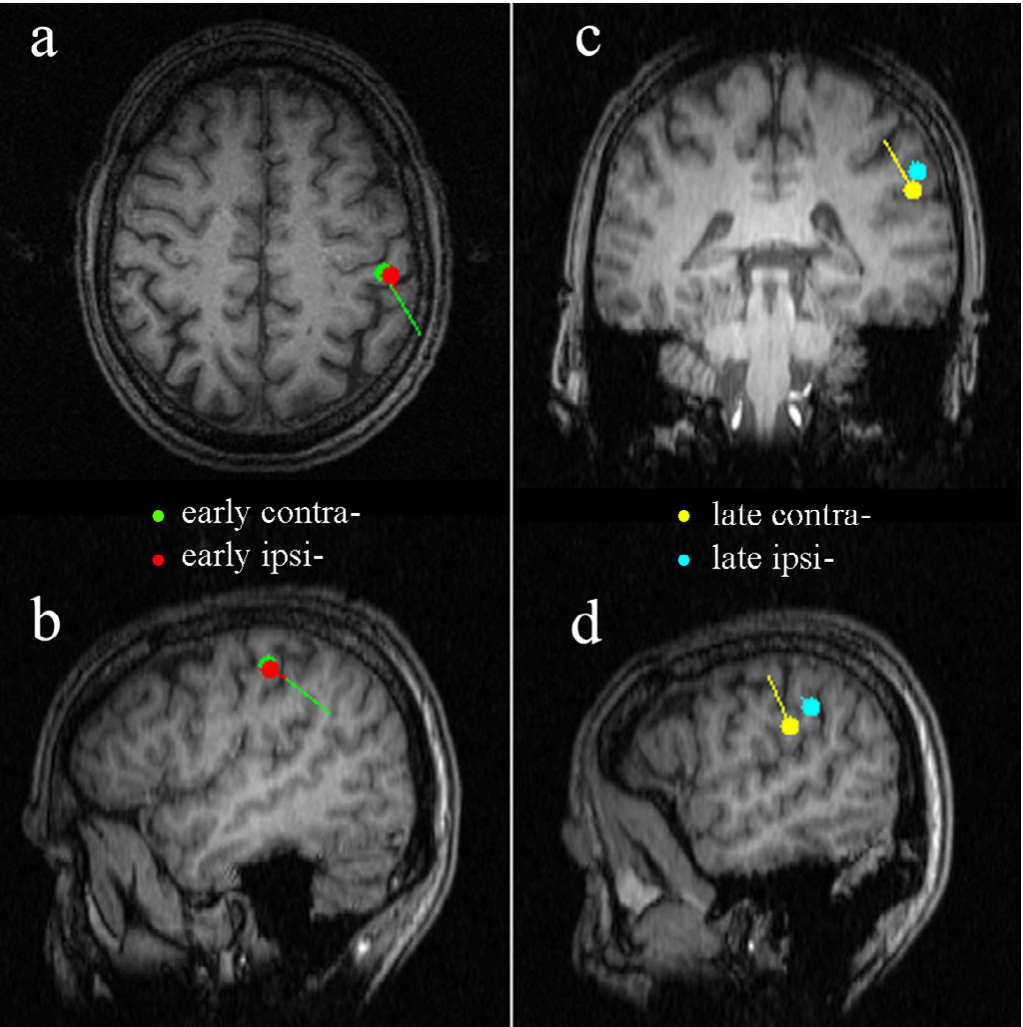
**Source localizations of responses to index finger stimulation alone**. Identified dipole sources of responses in the right hemisphere shown in Figure 1 are superimposed on this subject's MRI. The left horizontal (**a**) and sagittal (**b**) slices show the locations of early contralateral response (green dot in right anterior parietal field) to LD2 stimulation and early ipsilateral response (red dot) to RD2 stimulation. The coronal (**c**) and sagittal (**d**) slices in the right column show the locations of the late contralateral response (yellow dot in right S2) to LD2 stimulation and late ipsilateral response (cyan dot) to RD2 stimulation. The tails of those dots indicate dipoles' strength and direction.

In all subjects, dipoles were fit for both the early (30–70 ms) and late (70–130 ms) response windows. Figure 1 contains the actual contour plots of MEG sensor data and the plots of the averaged magnetic fields measured using 275 axial gradiometers from a single right-handed subject showing responses to index finger stimulation (average of 'deviants' alone at low rate, ISI = 2s). The left and right columns show responses to right index finger (RD2) and left index finger (LD2) stimulation, respectively. Identified dipole sources of responses in the right hemisphere were superimposed on this subject's MRI and are shown in Figure 2.

Interestingly, bilateral activation appeared during both early and late time periods in this subject for both LD2 and RD2 stimulation, though the early responses were of larger amplitude and shorter latency in the contralateral (e.g. the first peak at 45 ms in Figure 1e) versus the ipsilateral (e.g. the first peak at 51 ms in Figure 1f) hemisphere. The dipole direction and location of the ipsilateral (left hemisphere) response to LD2 stimulation were similar to those of the contralateral (left hemisphere) response to RD2 stimulation and likewise for the right hemisphere responses. The dipole directions tended to be mirror images in the two hemispheres. Dipole analysis and co-registration of the MEG localization information with the MRI scans verified that the anterior parietal area (the posterior wall of the central sulcus) was the primary contributor to both contralateral and ipsilateral early (30–70 ms) responses and that the S2 region (the upper bank of the Sylvian fissure) was the primary contributor to the late (70–130 ms) responses in this subject (Figure 2).

The average current dipole positions were specified in Talairach coordinates (x, y, z in mm): the early contralateral response dipoles in left and right hemispheres were (-49.2 ± 1.6, -19.2 ± 2.5, 45.8 ± 2.4) and (48.0 ± 1.4, -14.5 ± 1.4, 51.3 ± 3.7); the late contralateral response dipoles in left and right hemispheres were (-61.9 ± 3.7, -16.6 ± 3.4, 6.9 ± 3.8) and (56.2 ± 2.4, -5.4 ± 4.7, 13.9 ± 4.8), respectively. These localizations in Talairach space were consistent with the locations of anterior parietal fields and S2/PV in both hemispheres.

**Figure 3:**
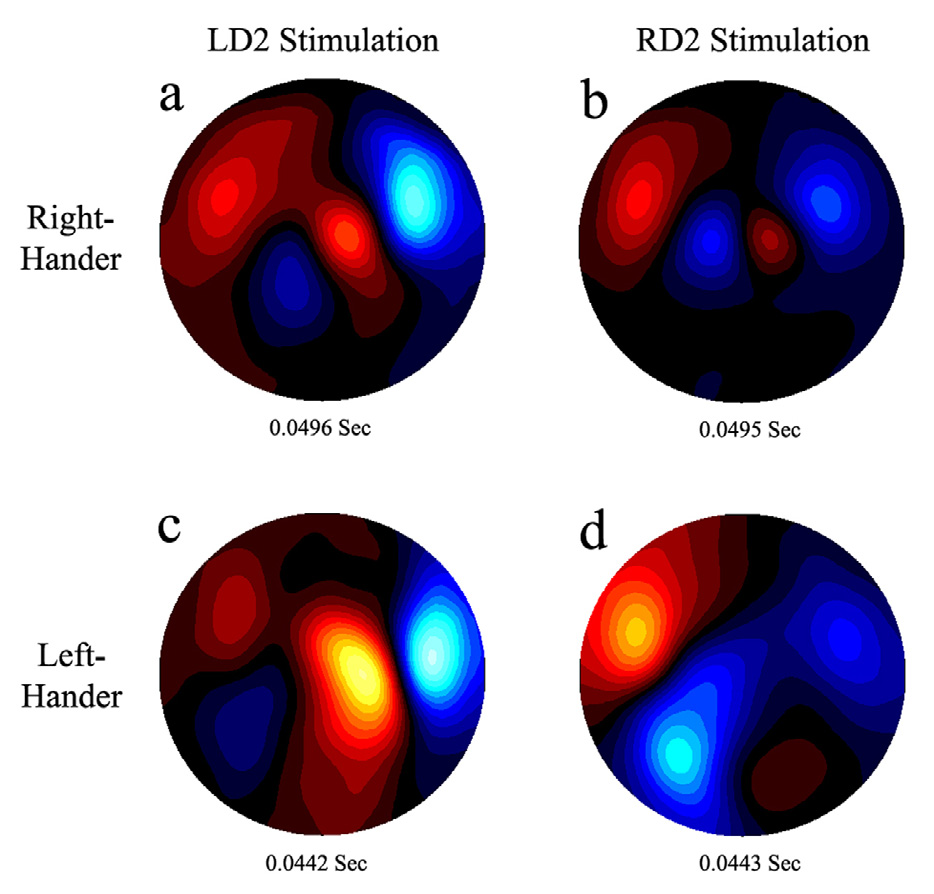
**Two ipsilateral early responses examples**. The top two averaged sensor data plots are from a right-handed subject, the bottom two are from a left-handed subject. Figures in the left column show the two subjects' sensor plots at the early ipsilateral (left hemisphere) response peak moment under the LD2 stimulation condition. The peak ipsilateral (left hemisphere) response is still weaker than the contralateral (right hemisphere) response, though the right hemisphere response is not at its peak at this time. Figures in the right column show similar results for RD2 stimulation. Both LD2 and RD2 stimulation elicited bilateral early responses in these two subjects.

Early ipsilateral responses for LD2 stimulation were found in 12/15 right- and 2/6 left-handed subjects, while 7/15 right- and 2/6 left-handed subjects showed early ipsilateral responses to stimulation of RD2. Figure 3 shows two more examples of early ipsilateral responses (one right-handed and one left-handed). The source of early ipsilateral responses was identified in anterior parietal fields. Late ipsilateral responses were recorded from all 21 subjects. Like the late contralateral responses, the late ipsilateral responses also originated from the upper banks of the Sylvian fissure, in the S2 region.

#### 2). Latency

The peak latencies of responses from a single subject (contralateral early and late responses, ipsilateral early and late responses) are shown in Figure 1. The early contralateral responses from both hemispheres of the same subject peaked at 45 ms, while late contralateral responses peaked at about 100 ms (Figure 1a, b and 1d, e). Early and late ipsilateral responses in the right hemisphere peaked at 60 and 120 ms (Figure 1a and 1c). The early ipsilateral responses in the left hemisphere peaked at 51 ms. Later responses, which looked more complex with multiple peaks, peaked around 110 ms (Figure 1d and 1f). Thus, both early and late ipsilateral response peaks appeared later than contralateral peaks in both hemispheres in this subject.

The average early and late contralateral response latencies for all subjects (right- and left-handed) were not significantly different across hemispheres (p > 0.05). The ipsilateral early (56.2 ± 6.6 ms) and late (116.6 ± 13.1 ms) responses appeared significantly later than contralateral early (46.8 ± 6.9 ms) and late (108.8 ± 12.5 ms) responses in all subjects. The mean delay of the early ipsilateral responses relative to the contralateral responses was 10.7 ± 6.1 ms (range: 3.5–24 ms), while the delay of the late ipsilateral responses relative to the contralateral responses was 12.7 ± 13.1 ms (range 3.0–30.8 ms). The mean difference between delays for the early versus late responses was not significant (p > 0.05).

#### 3). Amplitude

The subject's data from Figure 1 clearly show the early ipsilateral responses above the noise level (Figure 1c and 1f). The amplitudes of this subject's early ipsilateral responses were 47.3 and 35.8 fT (RMS), while the noise levels were 13.6 and 9.8 fT (RMS), in the left and right hemispheres, respectively. The average amplitude of the early ipsilateral response (37.9 ± 2.0 fT) across all subjects was significantly higher (p < 0.01) than the average noise level (14.5 ± 0.9 fT) based on the pre-stimulation period of 50 ms.

The average amplitude of early and late responses for all subjects did not show a significant difference between hemispheres (p > 0.05), nor was there a significant difference in amplitude of response for right- versus left-handers (p > 0.05). As the strength of early and late responses did not show a significant difference between hemispheres, the responses to left and right hand stimulation were combined for further analysis.

**Figure 4:**
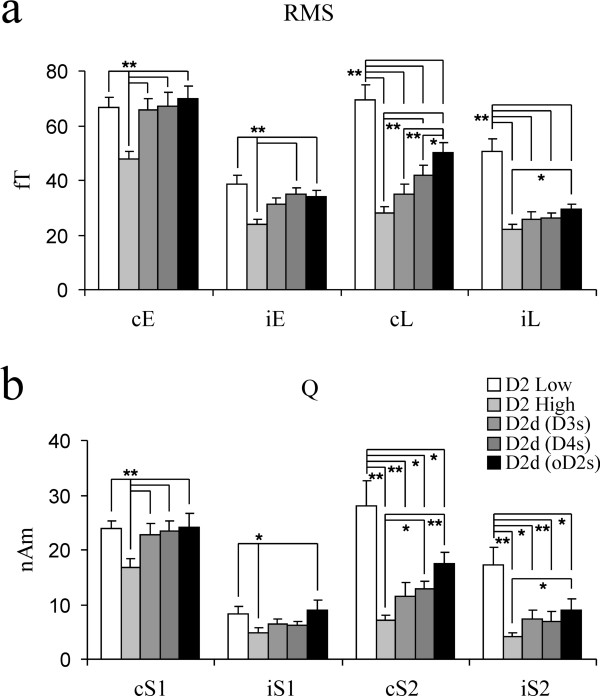
**Spatial integration**. Grand average of the peak sensor amplitude (**a**: RMS) and dipole moment (**b**: Q) values for early (anterior parietal area) and late (S2/PV) responses under different experimental conditions are compared. Asterisks indicate that the responses are significantly (*: p < 0.05; **: p < 0.01) different from others that are linked to it by the lines, using the Bonferroni post-hoc test. c, i, E and L stand for contralateral, ipsilateral, early and later response, respectively. S1 and S2 stand for anterior parietal areas and S2/PV respectively. D2 Low: D2 stimulation alone at low rate (condition 1 in Figure 6; mean ISI: 2s); D2 High: D2 stimulation alone at high rate (condition 2 in Figure 6; ISI: 0.33s); D2d (D3s): D2 deviant plus D3 standard stimuli (condition 3 in Figure 6; ISI: 0.33s); D2d (D4s): D2 deviant plus D4 standard stimuli (condition 4 in Figure 6; ISI: 0.33s); D2d (oD2s): D2 deviant plus opposite D2 standard stimuli (condition 5 in Figure 6; ISI: 0.33s).

The mean amplitude of the early response was significantly smaller (p < 0.01) for the early ipsilateral response (37.9 ± 2.0 fT) than for the early contralateral response (66.9 ± 3.3 fT). Similarly, the late ipsilateral response (50.4 ± 4.8 fT) was significantly weaker (p < 0.01) than the late contralateral response (69.3 ± 5.6 fT). Further, the mean amplitude of the early contralateral response (66.9 ± 3.3 fT) was not significantly different from the late contralateral response (69.3 ± 5.6 fT; p > 0.05), while the late ipsilateral response (50.4 ± 4.8 fT) was significantly stronger than the early ipsilateral response (37.9 ± 2.0 fT; p < 0.01; see Figure 4).

### Spatial integration between digit representations

#### 1). Latency

**Figure 5:**
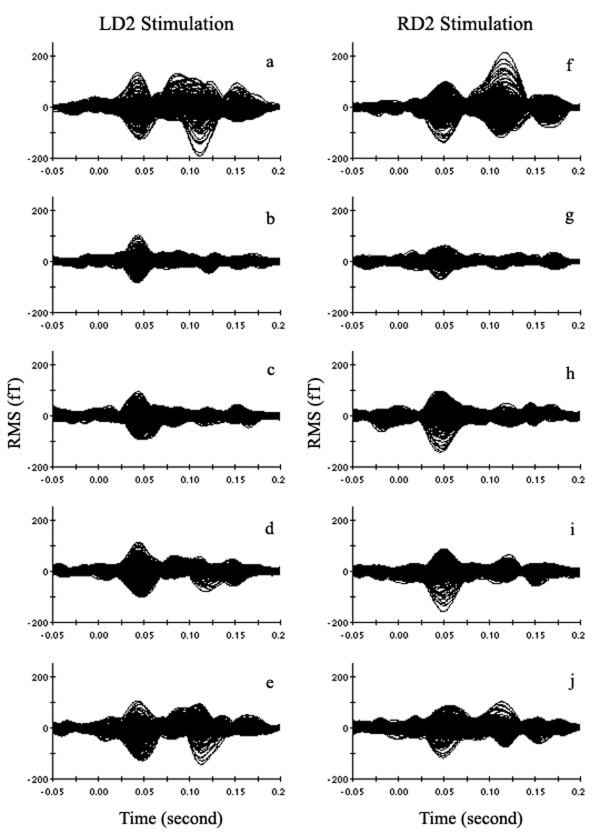
**An example of spatial integration obtained from a right-handed subject**. The waveforms show the time courses of the averaged magnetic field responses recorded from 275 sensors under the different conditions. The left and right columns show contralateral sensor response waveforms for LD2 and RD2 stimulation, respectively. **a **and **f**: D2 stimulation alone at low rate (condition 1 in Figure 6; mean ISI: 2s); **b **and **g**: D2 stimulation alone at high rate (condition 2 in Figure 6; ISI: 0.33s); **c **and **h**: D2 deviant plus D3 standard stimulation (condition 3 in Figure 6; ISI: 0.33s); **d **and **i**: D2 deviant plus D4 standard stimulation (condition 4 in Figure 6; ISI: 0.33s); **e **and **j**: D2 deviant plus opposite D2 standard stimulation (condition 5 in Figure 6; ISI: 0.33s).

Figure 5 is an example of the spatial interaction from a typical right-handed subject. This figure shows the averaged magnetic field responses recorded from all 275 sensors under the different stimulus conditions. Each line is the average over all trials for a given sensor. The left column in Figure 5 shows contralateral (right hemisphere) sensor waveforms for LD2 stimulation, and the right column shows the contralateral (left hemisphere) sensor waveforms for RD2 stimulation. The latencies of both early and late responses did not change under different stimulation conditions.

#### 2). Amplitude

In an example subject, low rate index finger stimulation (mean ISI: 2s) clearly elicited both early and later responses (Figure 5a and 5f). The response amplitude was markedly decreased for high rate stimulation (ISI: 0.33 s), and the late response (S2/PV) almost disappeared in both hemispheres (Figure 5b and 5g). The intervened standard stimuli (D3, D4 and opposite D2) suppressed the late response for D2 (deviant) stimulation in both hemispheres. There appeared to be the greatest attenuation for the same (D2) digit high rate stimulation compared to alternate standard digit (D3, D4 and opposite D2) stimulation, and greater attenuation for adjacent (D3) compared to non-adjacent (D4 and opposite D2) digit stimulation (Figure 5b–e and 5g–j). The standard stimuli reduced the amplitude of the early response for D2 (deviant) stimulation in some conditions for this subject (Figure 5c and 5j).

Grand averaged sensor amplitude (RMS; contralateral hemisphere only) and dipole moment (nAm) response values to deviant stimuli are displayed in Figure 4. Both sensor and moment values of early responses were statistically significantly smaller for high rate index finger stimulation (ISI: 0.33s) compared to low rate index finger stimulation (mean ISI: 2s). Both contralateral and ipsilateral early amplitude of responses to single finger stimulation (D2) were not significantly affected by stimulation of the other fingers (D3, D4 and opposite D2).

As with the early responses, sensor and moment values of the late responses were statistically significantly smaller for high rate index finger stimulation (ISI: 0.33s) compared to low rate index finger stimulation (mean ISI: 2s). The late response decreased more than the early response regardless of handedness (p < 0.01; also see Figure 5).

Further, the intervened standard stimuli (D3, D4, and opposite D2) significantly reduced the late contralateral and ipsilateral responses to deviant (D2) stimuli. In the contralateral hemisphere this effect was greater for the adjacent finger (D3) compared to the non-adjacent fingers (D4 or opposite D2). The attenuation of the late contralateral response was significantly greater for the same finger high rate stimulation (D2 alone at high rate) compared to non-adjacent finger (D4 or opposite D2) stimulation. For the ipsilateral response, there were no differences in amplitude reduction related to the location of intervened standard stimulation. Only opposite D2 stimulation differed from high rate D2 stimulation in the late ipsilateral response. The suppressive effect of opposite D2 stimulation was significantly stronger for the contralateral than the ipsilateral late response (Figure 4).

## Discussion

In summary, we examined the spatiotemporal integration across digit representations within anterior parietal somatosensory areas and S2/PV, as well as interhemispheric integration, using non-simultaneous tactile stimulation. We have shown 1) spatial integration of responses in S2/PV, with a decrease in interaction with the increase in separation of digit representations; 2) greater temporal integration of inputs within one digit representation in S2/PV versus anterior parietal fields; 3) both contra- and ipsilateral early responses in anterior parietal fields to cutaneous mechanical stimulation. The early ipsilateral response has a longer latency and lower amplitude than the early contralateral response; and 4) processing of cutaneous inputs is symmetrical across hemispheres. What follows is a discussion of these findings in light of previous work from human and non-human primates.

### Spatiotemporal integration between digit representations in S2/PV

The distance-dependence of spatial integration between digit representations in the S2/PV area was examined in this study. We found that the amplitude of the contralateral late responses to deviant stimuli (D2) decreased when standard stimuli (D3, D4 and opposite D2) were intervened. Like the distance-dependence of spatial integration in anterior parietal areas [11,14], the attenuation decreased with the increasing distance of separation between receptive fields, toward greater attenuation for adjacent fingers (D3) compared to non-adjacent fingers (D4 and opposite D2).

In agreement with previous MEG work on digit response interaction in S2/PV [8,9,11,12,26], our data show that the standard stimuli significantly attenuate responses in bilateral S2/PV to deviant (D2) stimulation. The pattern of spatial integration in S2/PV is consistent with the fact that neurons in S2 and PV have large receptive fields, frequently encompassing multiple digits [2,4-7,27], while cells in anterior parietal areas do not [28,29].

We also found significant temporal integration in the S2/PV region. The amplitude of the late response was smaller for high rate versus low rate index finger stimulation, and the late responses decreased more than the early responses with high rate stimulation, as previous SEP [30] and SEF [26,31-34] investigations have shown. This result is consistent with previous studies revealing the shorter recovery cycle or refractory period for neurons in anterior parietal areas than in S2 [35,36].

Some non-human primate studies have suggested serial information processing from anterior parietal areas to S2/PV [37,38], with tactile inputs from anterior parietal fields converging onto S2 [27,39]. However, other studies suggest hierarchical equivalence of anterior parietal areas and S2 for tactile processing in cats and new-world primates [40-42]. To date, the debate about serial versus parallel organization of somatosensory cortex in humans is unresolved. Both early [43] and late [44,45] concurrent activities in anterior parietal and lateral sulcal areas have been reported in humans. In contrast, a recent MEG study revealed that the onset latency of response following transcutaneous electrical stimulation was longer in S2/PV than in anterior parietal areas [46]. However, we cannot rule out the contribution of activity in S2/PV to early responses and anterior parietal areas to late responses. It is possible that both serial and parallel processing play a role in tactile perception.

Serial convergence may account for the spatial and temporal specialties in S2/PV. Unlike anterior parietal areas with restricted and defined neural receptive fields, the large, complex receptive fields within S2/PV may play an important role in integrating tactile inputs over space and time. For example, the digit representations in S2 and surrounding fields lack fine somatotopy, and some cells responded better to proprioceptive rather than cutaneous stimuli [27]. In addition, studies have found that 23% of cells in the S2 region were orientation tuned, often across multiple digit pads [27]. These receptive field properties, tuned for relatively long-range spatial and temporal integration, go some way towards explaining S2's purported involvement in functions like tactile object exploration and identification [47-50], bimanual coordination [2,4,6,7], and tactile learning and memory [49,50]. Consistent with this idea is evidence in humans that damage to parietotemporal cortices (including S2) causes impairment of tactile object recognition in the absence of basic somesthetic dysfunction [51,52].

### Spatiotemporal integration between digit representations in anterior parietal somatosensory areas

We found that the amplitude of responses in anterior parietal areas to deviant alone stimuli applied to a single digit was significantly lower at high rate (the ISI is also 0.33s) versus low rate (ISI: 2s) stimulation, as previous studies [26,30-34] have shown. This decrease of amplitude resulting from increased rate of stimulation was much smaller in anterior parietal areas than in S2/PV. However, the average amplitude of early responses in anterior parietal areas for deviant stimulation did not show a significant decrease when the standard stimuli were interleaved between deviants (ISI: 0.33s), though the amplitude decrease was seen in some cases. Previous work using non-simultaneous stimuli with long ISIs (1–4 s) also showed similar results [26,53]. However, an interaction between digit representations has been reported in anterior parietal areas when using simultaneous [11,14,15] or non-simultaneous stimuli with short (<100 ms) ISIs [13]. These findings are in line with the short recovery cycle time constant (~110 ms) for neurons in anterior parietal areas, which was estimated by fitting an exponential curve to SEF intensities for responses to electrical stimulation of the median nerve with different ISIs (100–500 ms) [36]. With a long ISI, the intervened standard inputs may arrive out of the recovery period of neurons in anterior parietal areas, with little effect on responses to deviant stimuli.

It is known that there is an inhibitory surround structure of receptive fields in monkey area 3b [54-58]. A three-component model has been proposed in which a lagged inhibitory surround overlaps the excitatory center and its one or more fixed inhibitory flankers. The fixed (as a spatial filter) and lagged (as a temporal filter) surround inhibitory components confer the spatial and temporal selectivity of neurons in anterior parietal areas [58]. The spatiotemporal property of inhibitory surround structure of neural receptive fields may account for the restricted neural receptive field and rapid recovery cycle in anterior parietal areas observed in the present study.

### Ipsilateral responses in anterior parietal areas

In the present investigation we clearly recorded early ipsilateral responses with high goodness-of-fit to single finger tactile stimulation (both right- and left-handers). Though the amplitude of the ipsilateral response was very weak in some cases, on average it was 2.5 times greater than the noise level. An ipsilateral response from anterior parietal field has also been observed in a small number of subjects in several previous studies [59-63] using MEG or EEG to measure responses to median nerve stimulations. A growing body of evidence from monkey studies [64,65] and human fMRI studies [66,67] also report ipsilateral responses in anterior parietal fields, suggesting that anterior parietal fields receive tactile input from the ipsilateral hand. Ipsilateral responses were relatively robust in our study, likely due to the cutaneous mechanical stimulation used in our experiment. Recent fMRI studies on both monkeys and humans have shown that responses from ipsilateral anterior parietal areas are more robust for mechanical versus median nerve electrical stimulation [64].

Single cell recordings from monkeys indicate that some neurons representing multiple digits in areas 2 and 5 have bilateral or ipsilateral receptive fields [68], and a few nociceptive neurons at the junction of areas 3b and 1 have large, bilateral receptive fields [69]. Thus the source of the early ipsilateral response is likely not only area 3b, but may also include input from cells in area 1, and possibly areas 2 and 5 as well. Recent evidence from electrophysiological recording in monkeys suggests that responses from ipsilateral anterior parietal fields are elicited by feedback rather than feedforward afferents [64], which may be related to the latency delay of the ipsilateral response observed in previous SEP [62] and the current MEG study. It has been suggested that transcallosal pathways may mediate responses from ipsilateral anterior parietal areas both in humans [59-61,67] and in monkeys [64,65]. However, anatomical evidence from macaque monkeys indicates that anterior parietal areas, in particular area 3b, have sparse or absent callosal connections for the hand representation [1,22,25]. Callosal connections become denser in the more caudal fields, from areas 3b to 1 and 2 [24]. Thus, transcallosal pathways mediating the early ipsilateral responses would be through these caudal areas, or S2/PV.

### Hemispheric differences

In addition, some previous functional imaging studies suggest functional dominance, or an increased source amplitude, in the left versus the right hemisphere in human primary [70-72] and secondary [73-77] somatosensory cortex. An expanded hand representation in left anterior parietal fields has also been proposed [72,78]. However, morphometric and cytoarchitectonic measurements show no lateralized differences in somatosensory cortices [79]. Psychophysical tests show no differences in spatial acuity between hands [80,81], although there does appear to be a left-hemisphere advantage for tactile processing of simultaneity judgment [82]. Furthermore, as in our study, previous somatosensory evoked potential studies show no difference in topography or response amplitude between the two hemispheres [83,84]. Thus, it is necessary to reassess the hemispheric asymmetry of somatosensory cortex.

The differences between studies may be due to the different types of stimuli used. The electrical stimulation used in previous studies showing asymmetry is not receptor specific, and elicits a more complex cortical activation. A recent MEG study showed that movement might be involved in the lateral asymmetry of somatosensory cortex, and the intensity of electrical stimulation often exceeded the motor threshold in previous studies [70-75,77]. Passive finger movement was found to evoke laterally asymmetrical responses in somatosensory cortex [76]. Thus, the increased response in left somatosensory cortex evoked by electric stimulation may reflect the lateral asymmetry of movement rather than tactile information processing.

## Conclusion

This human MEG study revealed that the bilateral spatiotemporal integration in S2/PV takes place over a large cortical area and over a long time period. Further, the strength of integration in this region is distance-dependent. The wide overlap of digit receptive fields in S2/PV might account for complex functions such as manual exploration and bimanual coordination, thought to be crucially dependent on S2/PV. In contrast, the properties of spatiotemporal integration in anterior parietal areas were different from those in S2/PV with significant temporal integration while spatial integration was reduced, which is consistent with the critical role of anterior parietal fields in spatial discrimination. The early ipsilateral response observed in over half of subjects suggests that anterior parietal fields do accept tactile inputs from the ipsilateral side of body. In addition, no tactile response difference between hemispheres indicated functional symmetry in human somatosensory cortex.

## Methods

### Subjects

SEFs were recorded in fifteen right-handed and six left-handed healthy adult subjects (10 male and 5 female right-handers; 2 male and 4 female left-handers; age range 22–40 years), using an Omega 2000 Whole-Cortex MEG System (CTF Systems Inc. Port Coquitlam, Canada; 275 DC SQUID first-order axial gradiometers). All subjects signed an informed consent as approved by the Committee on Human Research of the University of California, San Francisco. The Edinburgh inventory [85] was used to determine the direction and degree of handedness for each subject. The subjects were seated comfortably and maintained their head position during MEG testing in a magnetically shielded room. Subjects' hands were placed palm up on opposite armrests. The subjects closed their eyes, wore earplugs and were asked not to pay attention to the tactile stimulation.

### Stimuli

**Figure 6:**
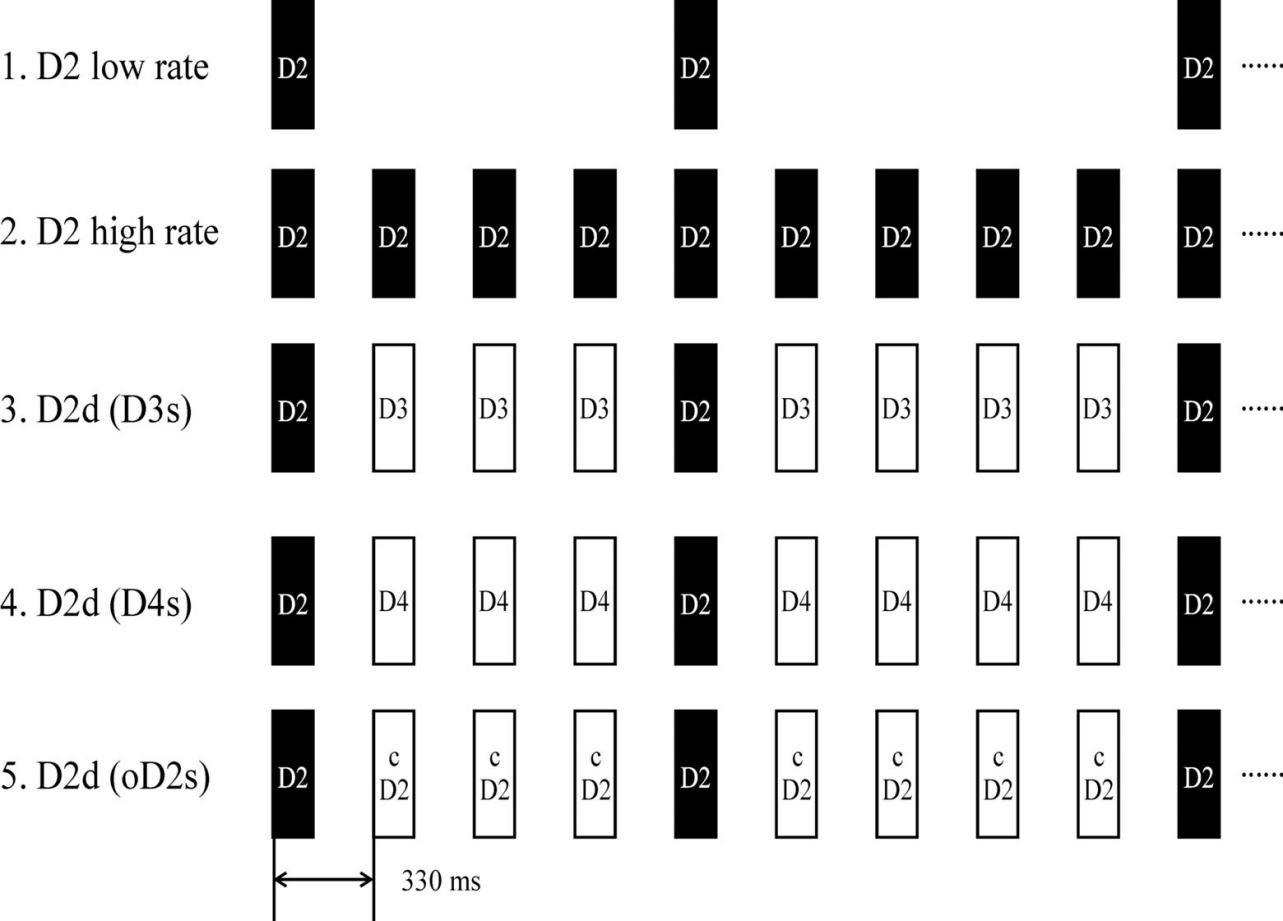
**Stimulus paradigm**. 1. Low rate (mean ISI; 2s) D2 stimulation (black bars) alone. 2. High rate (mean ISI: 0.33s) D2 stimulation alone. 3. Ipsilateral D3; 4. ipsilateral D4; 5. contralateral D2 as the standard stimulus respectively (white bars), with D2 as the deviant stimulus (black bars) in the digit mismatch paradigm. RD 3, 4 and LD 2 were used as standards with RD2 as deviants; LD 3, 4 and RD2 were used as standards with LD2 as deviants.

Tactile stimuli were pneumatically driven pulses (~140 ms duration) applied to the tips of the digits with balloon diaphragms. A digit oddball paradigm [9,16,30] was used to examine the representations of the index fingers (D2, infrequent deviant stimulus) while varying the location of the frequent standard stimuli across adjacent (D3), non-adjacent (D4) and contralateral (D2) digits. In this oddball paradigm, infrequent deviant stimuli interspersed with frequent standard stimuli were presented. The intensity of all stimuli was well above detection threshold at 17 PSI (pounds per square inch). The location of the standard was changed between blocks and the order (including deviant alone block) was randomized across subjects. In each block of 600–900 trials with an ISI of 0.33s and a jitter of 10 ms, standard and deviant stimuli were presented at probabilities of 0.83 and 0.17 respectively. Deviants were always followed by 3–7 standards to allow for an adaptation to the standard. To compare the tactile response to single finger stimulation in different hemispheres, deviants were also presented alone at two rates: one equal to that for deviants (mean ISI: 2 s) and the other equal to that for the compound of deviants and standards (mean ISI: 0.33s) in the mismatch paradigm (Figure 6).

### Data recording and analysis

Data were collected at a sample rate of 1200 Hz. The filtering passband for data analysis was 2–40 Hz. About 100 artifact-free trials for deviant stimuli (D2) were averaged in each test block. Head position relative to the MEG sensors was determined before and after each test block by means of three small coils placed at landmark sites (nasion, left and right preauricular points).

MEG data was analyzed using an equivalent current dipole (ECD) embedded in a spherical conducting medium. The locations of index finger representations in somatosensory cortex were determined using a dipole fit with contralateral index finger stimulation alone. SEFs in somatosensory cortex peaking in the time window up to 200 ms following stimulus onset were analyzed. The early (30–70 ms) response was analyzed for activation in anterior parietal fields; and the late response (70–130 ms) was analyzed for activation of S2/PV [11,26]. Sensors recording from the hemisphere contralateral to the stimulated index finger were chosen to determine the ECD of the most dominant source. The position and orientation of the ECD corresponding to the early response were first found and fixed; then another dipole corresponding to the late response was added with the early one fixed, then was fitted and fixed successively. Only sources with high goodness of fit (> 85%) were accepted. Dipoles matching anterior parietal fields and S2/PV in each hemisphere were identified and fixed based on contralateral index finger stimulation, and they were used for analysis of data from other stimulation conditions. The response latencies, amplitudes (root-mean-square value, RMS) of each hemisphere and dipole moments (Q value) for all of four dipole locations were estimated for the different stimulation conditions based on peaks within the early and late time periods.

Eight of the subjects were also scanned using a 1.5T MRI scanner (GE Medical System, Milwaukee, WI) to acquire a 3D structural image (flip angle = 40°, TR = 27 ms, TE = 6 ms, FOV = 240 × 240 mm, 1.5 mm slice thickness, 256 × 256 × 124 pixels). Three fiducials were placed on the subject at the same three locations as the localizing coils in MEG. This information was used to coregister the MEG data to the MRI image. Each subject's structural MRI was normalized to MNI space using Neurodynamic Utility Toolbox for MEG [86]. Then the MNI coordinates of dipole positions were converted to Talairach coordinates, using a non-linear transform [87].

Paired Student t-tests were used to assess significant difference between condition pairs. In cases where the number of conditions exceeds two, repeated measures ANOVAs were used for assessing statistical significance between responses across different stimulation conditions. In these cases, Bonferroni post-hoc tests were used to assess significance between specific condition pairs, whereby after correction for multiple comparisons a significance threshold of either p < 0.01 or p < 0.05 was used. Data are presented as mean values ± standard error of the mean throughout.

## List of abbreviations

PV: parietal ventral area

MEG: magnetoencephalography

SEFs: somatosensory evoked fields

ECD: equivalent current dipole

RMS: root mean square

Q: dipole moment

LD2: left index finger

RD2: right index finger

## Authors' contributions

ZZ, EAD, DJM and SSN designed the study. ZZ and EAD drafted the manuscript. JMZ, DJM and SSN helped to draft the manuscript. ZZ and JMZ recorded and analyzed MEG and MRI data. All authors read and approved the final manuscript.
